# Point-of-care Ultrasound Diagnosis of Cholecystitis vs. Adenomyomatosis

**DOI:** 10.5811/cpcem.2019.2.40798

**Published:** 2019-03-27

**Authors:** Esther Kim, Rame Bashir, Shadi Lahham

**Affiliations:** University of California-Irvine, Department of Emergency Medicine, Orange, California

## CASE PRESENTATION

A previously healthy 26-year-old male presented with three days of right upper quadrant (RUQ) pain, worsened with food. Physical exam demonstrated a focal RUQ peritonitis, or positive Murphy’s sign, with no rebound or guarding. Vital signs were stable, and labs showed no leukocytosis or metabolic derangements. Point-of-care ultrasound (POCUS) demonstrated a stone in the gallbladder neck, 4.6 millimeters anterior wall thickness, but no pericholecystic fluid (Video). Surgery was consulted and the patient was offered outpatient follow-up for biliary colic with adenomyomatosis. He returned to the emergency department (ED) the following day with persistent pain and underwent cholecystectomy for cholecystitis.

## DISCUSSION

While gallstones are the most common cause of cholecystitis, 80% of patients with gallstones are asymptomatic and less than 3% develop acute cholecystitis.^1^ While the presence of RUQ pain, fever, and leukocytosis are the clinical criteria for cholecystitis, the diagnosis requires imaging. A sonographic Murphy’s sign is the most sensitive sign of cholecystitis (86%–88%); however, pericholecystic fluid and gallbladder wall thickening are nonspecific.^2^–^3^

Gallbladder wall thickening is associated with many conditions including adenomyomatosis. Adenomyomatosis is an idiopathic hyperproliferation of the gallbladder wall and is found in 9% of people.^4^ Characteristically, bile- or calcium-filled sinuses, or Rokitansky-Aschoff sinuses (RAS), as well as “comet tail” artifacts are diagnostic.^5^ Adenomyomatosis has been associated with gallstones; however, this association was not significant.^5^ It is not associated with pericholecystic fluid. Although most patients with adenomyomatosis are asymptomatic and do not require intervention, caution should be taken to differentiate it from acute cholecystitis. Contrast-enhanced ultrasound or magnetic resonance imaging should be used if initial ultrasound is equivocal.^5^

Adenomyomatosis and cholecystitis are not mutually exclusive and the patient’s clinical picture is paramount. Absence of pericholecystic fluid, leukocytosis, and fever in the presence of RAS, and the presence of a gallstone (as in our patient) should not lower our threshold for emergent cholecystectomy, as symptomatic RAS, even in the absence of stone, is indication for surgery.^6^ Additionally, other differential diagnoses should be considered in the context of persistent RUQ pain and a thickened gallbladder wall. For instance, rare mucous-producing gallbladder neoplasms have been associated with cystic spaces resembling RAS.^5^

In summary, ED providers should maintain a low threshold for emergent cholecystectomy for patients with a positive Murphy’s sign with ultrasonographic evidence of stone. In this way, POCUS can help minimize both unnecessary cholecystectomy and delays in patient care.

**CPC-EM Capsule**

What do we already know about this clinical entity?

*Adenomyomatosis is an idiopathic hyperproliferation of the gallbladder wall. The presence of bile or calcium filled sinuses called Rokitansky-Aschoff sinuses are diagnostic*.

What is the major impact of the image(s)?

*Like cholecystitis, adenomyomatosis may present with gallbladder wall thickening on imaging but is often asymptomatic and does not require intervention. Gallbladder wall thickening in the absence of right upper quadrant pain, fever, leukocytosis and pericholecystic fluid should be further evaluated to avoid unnecessary cholecystectomy*.

How might this improve emergency medicine practice?

*Differentiating adenomyomatosis from acute cholecystitis using a thorough history, physical exam, and diagnostic imaging can minimize unnecessary intervention and morbidity*.

## Supplementary Information

Video Right upper quadrant ultrasound demonstrating a stone in gallbladder neck, and anterior gallbladder wall measuring 4.6 millimeters with no pericholecystic fluid. Findings are also consistent with adenomyomatosis as evidenced by the Rokitansky-Aschoff sinuses in wall and “comet tail” artifacts (red arrows).^5^
